# Cancer-Specific Survival Outcome in Early-Stage Young Breast Cancer: Evidence From the SEER Database Analysis

**DOI:** 10.3389/fendo.2021.811878

**Published:** 2022-01-18

**Authors:** Rui Liu, Zhesi Xiao, Daixing Hu, Haojun Luo, Guobing Yin, Yang Feng, Yu Min

**Affiliations:** ^1^Department of Oncology, The Second Affiliated Hospital of Chongqing Medical University, Chongqing, China; ^2^Department of Breast and Thyroid Surgery, The Second Affiliated Hospital of Chongqing Medical University, Chongqing, China

**Keywords:** breast cancer, nomogram, cancer-specific survival, early-stage, SEER

## Abstract

**Background:**

Young women with breast cancer are determined to present poorer survival compare with elderly patients. Therefore, identifying the clinical prognostic factors in young women with early-stage (T_1-2_N_0-1_M_0_) breast cancer is pivotal for surgeons to make better postoperative management.

**Methods:**

The clinicopathological characteristics of female patients with early-stage breast cancer from the Surveillance, Epidemiology, and End Results program between Jan 2010 and Dec 2015 were retrospectively reviewed and analyzed. Univariate and multivariate Cox regression analyses were used to determine the potential risk factors of cancer-specific survival in young women with early-stage breast cancer. The nomogram was constructed and further evaluated by an internal validation cohort. The Kaplan-Meier survival curves were used to estimate cancer-specific survival probability and the cumulative incidence.

**Results:**

Six variables including race, tumor location, grade, regional lymph node status, tumor subtype, and size were identified to be significantly associated with the prognosis of young women with early-stage breast cancer during the postoperative follow-up. A nomogram for predicting the 3-, 5- year cancer-specific survival probability in this subpopulation group was established with a favorable concordance index of 0.783, supported by an internal validation cohort with the AUC of 0.722 and 0.696 in 3-, 5- year cancer-specific survival probability, respectively.

**Conclusions:**

The first predictive nomogram containing favorable discrimination is successfully established and validated for predicting the 3-, 5- year cancer-specific survival probability in young women with early-stage breast cancer during the postoperative follow-up. This model would help clinicians to make accurate treatment decisions in different clinical risk population.

## Introduction

Breast cancer has become the most frequently diagnosed malignancy and one of the leading causes of cancer-specific death in China and around the world (1–4), with a female predominance. Although young women (usually refers to <40 years) made up only a limited proportion of breast cancer, a similar increasing prevalence was also observed in this subpopulation (5, 6). Among adolescents and young adults, the overall cancer mortality declined over the past few decades by 1% annually across age and sex groups. However, the rates were stable in young female patients aged between 30-39 years because of a flattening of declines in female breast cancer (5). According to the latest report from the American Cancer Society (5), the breast cancer incidence rate was 0.1, 5.7, and 46.6 per 100,000 population among young women aged between 15-19 years, 20-29 years, and 30-39 years, respectively. Furthermore, approximately 43% and 7% of young women were diagnosed at the regional or distant stage and the breast cancer-specific death reached 22% among women aged 15-39 years.

Compared with elderly women, young patients, accounting for a relatively small number of breast cancer, were more likely to present aggressive subtypes and advanced disease, and the survival and outcomes were worse (5-year relative survival rate comparison: 86% vs 91% in elderly women) (5). Consequently, the strategies of treatment and prevention for young breast cancer have gradually aroused wide attention (7–11). Notably, in one study from China, young patients with breast cancer were more likely to have larger tumor size, poorer differentiation grade, a higher proportion of triple-negative breast cancer (TNBC), and more advanced stage, when compared with elder patients (11).

Currently, only a small number of listed studies were focused on investigating the risk factors for predicting the clinical outcomes in young women with breast cancer (10, 12). For instance, based on the clinicopathological features of the large-scale population, Billena et al. (9) determined that tumor size, hormone receptor status, surgery, adjuvant therapies, lymph node status, and race were independent predictive factors for overall survival (OS) in young female breast cancer. To the best of our knowledge, however, neither systematic attempts have ever been made to explore the risk factors for predicting the cancer-specific survival (CSS) in young women with early-stage (T_1-2_N_0-1_M_0_) breast cancer nor develop prognostic nomograms. Therefore, in this present study, we aim to investigate the independent prognostic factors for CSS of young women with early-stage breast cancer and further construct and validate a visualized predictive model for clinicians to identify the patients with high risk and make better-individualized management (relatively more aggressive treatment approaches) for these patients.

## Materials and Methods

### Data Source

The data we analyzed were extracted from the Surveillance, Epidemiology, and End Results (SEER) 18 registry research database, which represented approximately 28% of the U.S. population and included various ethnic groups. For this study, we signed the SEER research data agreement to access SEER information with the reference of the username “10189-Nov2020”. Data were collected following the approved guideline. Data analysis from this database is considered to be non-human subjects by the Office for Human Research Protection as part of the US Department of Health and Human Services, because patient data was anonymized and publicly available. For these reasons, the need for ethics approval was waived by The Second Affiliated Hospital of Chongqing Medical University Ethics Committee.

Patients who met the following criteria were included: 1) young female patients between the age of 18 and 40 years; 2) diagnosis year between 2010 and 2015; 3) the diagnosis of breast cancer was confirmed by histopathology; 4) TNM stage was derived from the AJCC staging system 7^th^ edition. The excluding criteria: 1) No regional lymph node examined; 2) Patients were diagnosed with distant metastasis; 3) Patients coexisted with one or more cancers; 4) Incomplete medical records.

### Variables Evaluation and Definition

After excluding the unqualified cases, there were 7203 young female patients with invasive breast cancer enrolled in this retrospective cohort study. The included patients were randomly divided into a training group and validating group at a ratio of 7:3. The following clinicopathological characteristics were collected and transformed into categorical variables: age (>18 and <40 years), race (Hispanic, non-Hispanic White, non- Hispanic Black, non-Hispanic Asian or Pacific Islander, and non-Hispanic American Indian/Alaska Native), laterality (right and left origin of primary), stage (I and II derived from AJCC staging system 7^th^ edition), grade (well differentiated, moderately differentiated, poorly differentiated), location (central, outer, inner, overlapping and axillary of breast), histological type (infiltrating ductal carcinoma (IDC), infiltrating lobular carcinoma (ILC), and infiltrating ductal mixed lobular carcinoma (IDLC)), ICD-O-3 codes: 8500/3, 8503/3, 8507/3, 8500/3, 8520/3, 8521/3, and 8522/3), regional lymph node status (N0: no regional lymph node metastasis; N1: 1-3 axillary lymph nodes metastasis and/or internal mammary lymph node metastases), the number of regional nodes examined and positive nodes, breast cancer subtype (Luminal A: hormonal receptor (HR)+/Her-2-, Luminal B: HR+/Her-2+, Triple-negative: HR-/Her-2-, Her-2 enriched: HR-/Her-2 2+), tumor size (T_1_: >0mm and ≤20mm, T_2_: >20mm and ≤50mm), cause-specific death, and survival months (more than 0 days of survival).

### Statistical Analysis

The primary endpoint of this study was breast cancer-specific death during the follow-up. A two-tail P-value of <0.05 was defined as the criterion for variable deletion when performing backward stepwise selection. The development and validation of the nomogram, calibration curve, and Kaplan-Meier analysis were based on the results of the multivariate Cox regression analysis using the “survival”, “rms”, “survminer”, and “foreign” packages of the R software (R Foundation, Vienna, Austria, version 3.5.2, http://www.r-project.org). The area under the receiver (AUC) operating characteristic (ROC) curve and the Harrell’s C-index (an important indicator to estimate the discrimination capability of each prognostic model and to compare their prognostic performance) (13) are conducted to assess the feasibility of the present nomogram.

## Results

### Clinicopathological Characteristics of Patients

From the SEER database, a total of 7203 young female patients aged between 18 and 40 years old with early-stage breast cancer were ultimately included in this study and further randomized into training (5042 cases) and validating (2161 cases) cohorts at a ratio of 7:3. A majority of patients were white which accounted for 53.6% in the training cohort and 54.4% in validating cohort, respectively. Besides, the foremost proportion of differentiated grade was in grade III (poorly differentiated) with a ratio of 57.4%, whereas grade I (well-differentiated) and II (moderately-differentiated) were presented in only 8.1% and 34.9% of patients. Notably, there were more than half of patients diagnosed with breast cancer at T_2_ tumor size, and approximately 40% of patients suffered from regional lymph node metastasis. The median and average follow-up months in the training cohort were 52 and 48 months (a range of 0- 65 months), respectively. The specific demographic and clinical characteristics of the patients in the training and validation datasets were summarized in **Table 1**.

**Table 1:** Clinicopathological characteristics of young women with early-stage breast cancer.

Variables	No. (%) of Patients		
	Initial Cohort (n = 7203)	Training Cohort (n = 5042)	Validating Cohort (n = 2161)
Age (years)			
Mean ± SD	34.8 ± 3.8	34.8 ± 3.7	34.8 ± 3.8
Race			
Hispanic	1369 (19.0)	951 (18.9)	418 (19.3)
White	3879 (53.8)	2704 (53.6)	1175 (54.4)
Black	962 (13.4)	687 (13.6)	275 (12.7)
^※^Other	993 (13.8)	700 (13.9)	293 (13.5)
Location			
central	288 (4.0)	209 (4.1)	79 (3.6)
outer	1548 (21.5)	1089 (21.6)	459 (21.3)
inner	3552 (49.3)	2509 (49.8)	1043 (48.3)
^¶^other	1815 (25.2)	1235 (24.5)	580 (26.8)
Grade			
well	584 (8.1)	387 (7.7)	197 (9.1)
moderate	2516 (34.9)	1762 (34.9)	754 (34.9)
poor	4103 (57.0)	2893 (57.4)	1210 (56.0)
Histology			
IDC	6786 (94.2)	4765 (94.5)	2021 (93.5)
ILC	157 (2.2)	108 (2.1)	49 (2.3)
IDLC	260 (3.6)	169 (3.4)	91 (4.2)
Laterality			
right	3604 (50.0)	2556 (50.7)	1048 (48.5)
left	3599 (50.0)	2486 (49.3)	1113 (51.5)
^￥^Stage			
IA	2472 (34.3)	1692 (33.6)	780 (36.1)
IB	241 (3.3)	180 (3.6)	61 (2.8)
IIA	2703 (37.5)	1924 (38.2)	779 (36.0)
IIB	1787 (24.8)	1246 (24.7)	541 (25.0)
Tumor size (mm)			
T_1a_	299 (4.1)	217 (4.3)	82 (3.8)
T_1b_	617 (8.6)	428 (8.5)	189 (8.7)
T_1c_	2571 (34.9)	1771 (35.12)	800 (37.0)
T_2_	3716 (51.6)	2626 (52.1)	1090 (50.4)
Lymph node status			
N_0_	4401 (61.1)	3072 (60.9)	1329 (61.5)
N_1_	2802 (38.9)	1970 (39.1)	832 (38.5)
ER status			
negative	1989 (27.6)	1405 (27.9)	584 (27.0)
positive	5214 (72.4)	3637 (72.1)	1577 (73.0)
PR status			
negative	2629 (36.5)	1856 (36.8)	773 (35.8)
positive	4574 (63.5)	3186 (63.2)	1385 (64.1)
Her-2 status			
negative	5473 (76)	3825 (75.9)	1648 (76.3)
positive	1730 (24)	1217 (24.1)	513 (23.7)
Subtype			
Luminal A	4026 (55.9)	2802 (55.6)	1224 (56.6)
Luminal B	1332 (18.5)	939 (18.6)	393 (18.2)
TNBC	1447 (20.1)	1023 (20.2)	424 (19.6)
Her-2 enriched	398 (5.5)	278 (5.5)	120 (5.5)

**^※^**Other: defined as the non-Hispanic Asian/Pacific Islander and American Indian/Alaska Native; ^¶^other: axillary and overlapping of the breast;**^￥^**Stage: derived from the AJCC 7^th^ guideline. IDC, invasive ductal carcinoma; ILC, invasive lobular carcinoma; IDLC, invasive ductal mixed with lobular carcinoma; ER, estrogen receptor; PR, progesterone receptor; TNBC, triple-negative breast cancer; Her-2, human epidermal growth factor receptor-2; CSS, cancer-specific survival.

### Univariate and Multivariate Analyses of the Risk Factors of CSS

To screen out the potential independent risk factors of CSS in young women with early-stage breast cancer during the postoperative follow-up, only significant factors from univariate Cox regression analysis were further applied into multivariate Cox regression analysis (**Table 2**). During the univariate Cox regression analysis, race (*p*<0.001), tumor location (*p*=0.028), grade (*p*<0.0001), lymph node status (*p*<0.0001), subtype (*p*<0.0001), and tumor size (*p*<0.0001) were identified to be the independent predictive factors. The black race (hazard ratio (HR)=1.52, 95% confidence interval (CI): 1.05- 2.20, p=0.001), inner location (HR=2.36, 95%CI: 1.02- 5.46, p=0.003), moderately and poorly grade (HR=8.86, 95%CI: 1.22- 64.26, HR=14.04, 95%CI: 1.95- 101.08, respectively, p<0.0001), lymph node metastasis (HR=2.233, 95%CI: 1.81- 3.01, P<0.0001), TNBC (HR=2.28, 95%CI: 1.71- 3.03, p<0.0001), and T_2_ (HR=2.09, 95%CI: 1.56- 2.81, p<0.0001) were regarded as the independent risk factors of CSS in these patients.

**Table 2:** Univariate and multivariate Cox regression analyses of predictive variables correlated with CSS in young women with early-stage breast cancer.

Variables	Subgroup	Univariable		Multivariable	
		Hazard Ratio	*P*	Hazard Ratio	*P*
Race	Hispanic	Reference	<0.001	Reference	0.001
	White	0.74 (0.54- 1.03)		0.89 (0.64- 1.24)	
	Black	1.60 (1.10- 2.31)		1.52 (1.05- 2.20)	
	^※^Other	0.52 (0.32- 0.87)		0.62 (0.37- 1.02)	
Location	^&^central	Reference	0.028	Reference	0.003
	inner	2.38 (1.03- 5.48)		2.36 (1.02- 5.46)	
	outer	1.63 (0.71- 3.70)		1.41 (0.62- 3.23)	
	^¶^other	1.97 (0.85- 4.55)		1.97 (0.85- 4.57)	
Grade	well	Reference	<0.0001	Reference	0.001
	moderately	10.90 (1.50- 78.94)		8.86 (1.22- 64.26)	
	poorly	27.89 (3.91- 198.95)		14.04 (1.95- 101.08)	
Lymph node status	N_0_	Reference	<0.0001	Reference	<0.0001
	N_1_	2.21 (1.72- 2.83)		2.233 (1.81- 3.01)	
Histology	IDC	Reference	0.431	/	
	ILC	0.54 (0.17- 1.68)			
	IDLC	0.74 (0.35- 1.57)			
Subtype	Luminal A	Reference	<0.0001	Reference	<0.0001
	Luminal B	0.49 (0.30- 0.81)		0.40 (0.24- 0.66)	
	TNBC	3.11 (2.40- 4.04)		2.28 (1.71- 3.03)	
	Her-2	1.25 (0.70- 2.23)		0.97 (0.54- 1.74)	
Size	T_1_	Reference	<0.0001	Reference	<0.0001
	T_2_	3.14 (2.35- 4.19)		2.09 (1.56- 2.81)	

**^※^**Other: defined as the non-Hispanic Asian/Pacific Islander and American Indian/Alaska Native; **^&^**central: central portion of breast combined with nipple; ^¶^other: axillary and overlapping of the breast. IDC, invasive ductal carcinoma; ILC, invasive lobular carcinoma; IDLC, invasive ductal mixed with lobular carcinoma; TNBC, triple-negative breast cancer; Her-2, human epidermal growth factor receptor-2; T_1_, 0mm≥maximum diameter <20mm; T_2_, 20mm≥maximum diameter <50mm. Bold values indicate statistical significance (p<0.05).

**Figure 1:**
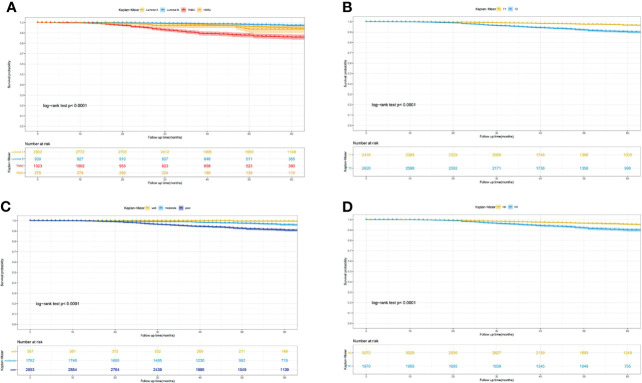
Kaplan-Meier curves for predicting the 3-, 5- year CSS of young women with early-stage breast cancer. **(A)** different molecular subtype; **(B)** tumor size; **(C)** differentiation grade; **(D)** lymph node status. CSS, cancer-specific survival.

**Figure 2:**
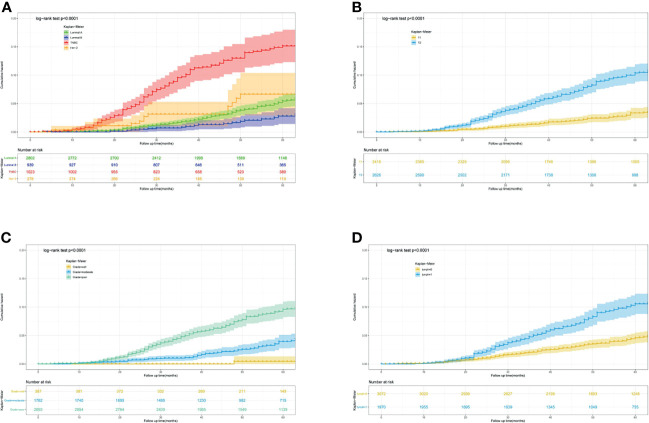
Predicting the 3-, 5- year cumulative hazard of cancer-specific death risk in young women with early-stage breast cancer. **(A)** different molecular subtype; **(B)** tumor size; **(C)** differentiation grade; **(D)** lymph node status.

Furthermore, to actuarially estimate the survival probability and cumulative hazard in patients with different variables after surgery, we selected four factors (p ≤ 0.001) from multivariate analysis in Cox proportional hazard models to plot the Kaplan–Meier survival curves and cumulative hazard. Specifically, a significant decrease in cumulative survival rate was observed in TNBC patients, compared with Luminal A, Luminal B, and Her-2 enriched subtypes (3- year CSS: 92.8% vs 98.8% vs 99.3% vs 96.9%; 5- year CSS: 85.9% vs 94.7% vs 97.3% vs 93.6%, *p*<0.0001, **Figure 1A**). Similarly, tumor size (T_1_ vs T_2_: 3- year CSS: 99.0% vs 96.3%; 5- year CSS: 96.7% vs 90.1%, *p*<0.0001, **Figure 1B**), differentiation grade (well vs moderately vs poorly: 3- year CSS: 99.6% vs 99.0% vs 96.4%; 5- year CSS: 99.6% vs 96.1% vs 90.9%, *p*<0.0001, **Figure 1C**), and regional lymph node status (N_0_ vs N_1_: 3- year CSS: 98.4% vs 96.3%; 5- year CSS: 95.5% vs 90.0%, *p*<0.0001, **Figure 1D**) were all associated with the cumulative survival probability. Moreover, the cumulative incidence of cancer-specific death increased to 0.15 in TNBC (**Figure 2A**), 0.1 in T_2_ (**Figure 2B**), 0.1 in poorly differentiated grade (**Figure 2C**), and 0.11 in N_1_ (**Figure 2D**), respectively.

### Predictive Nomogram Construction and Validation

**Figure 3:**
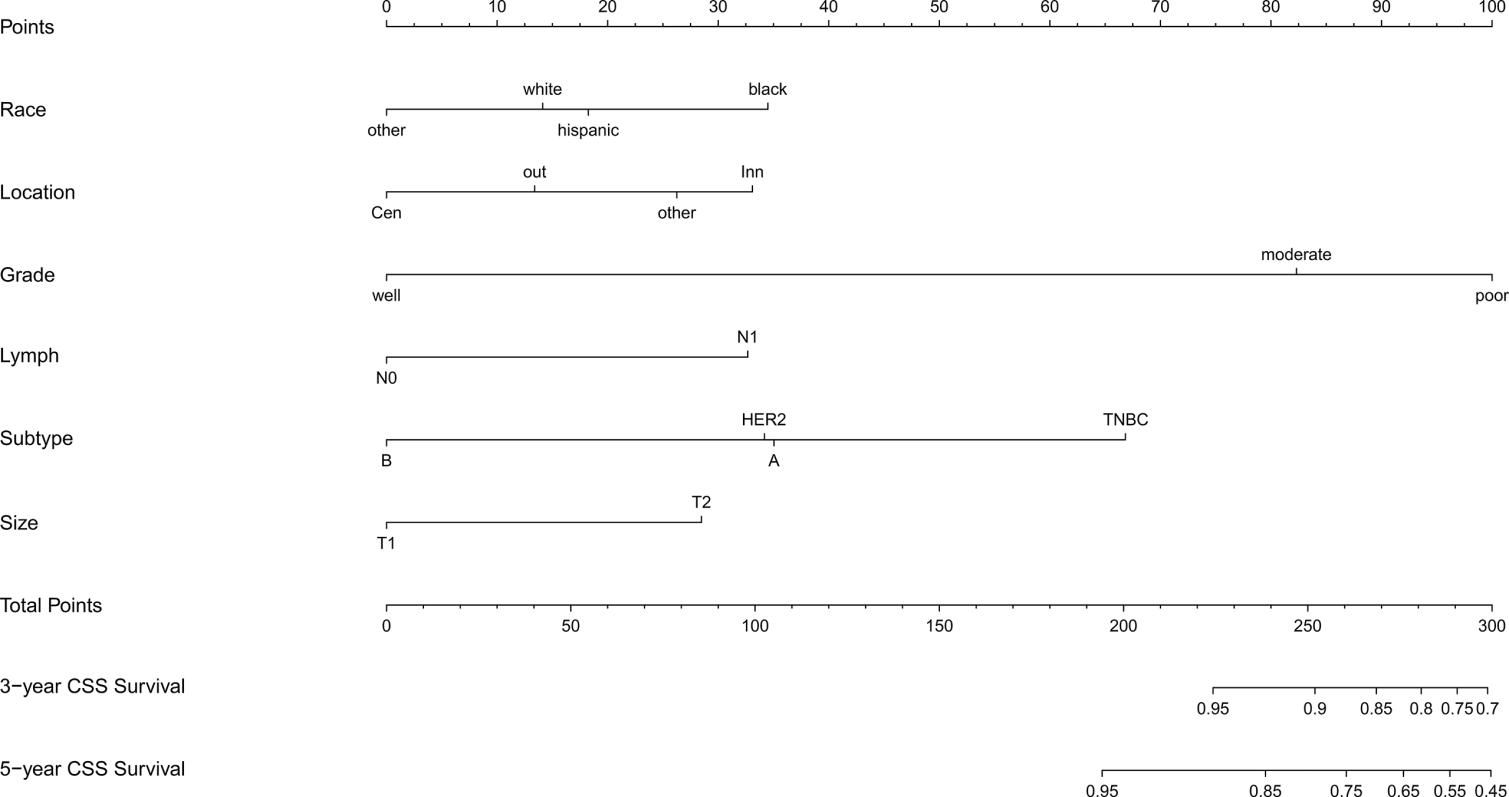
Nomogram for predicting the 3-,5- year CSS in young women with early-stage breast cancer. Note: other: defined as the non-Hispanic Asian/Pacific Islander and American Indian/Alaska Native; CSS, cancer-specific survival; A, Luminal A; B, Luminal B; TNBC, triple-negative breast cancer; HER2, Her-2 enriched.

**Figure 4:**
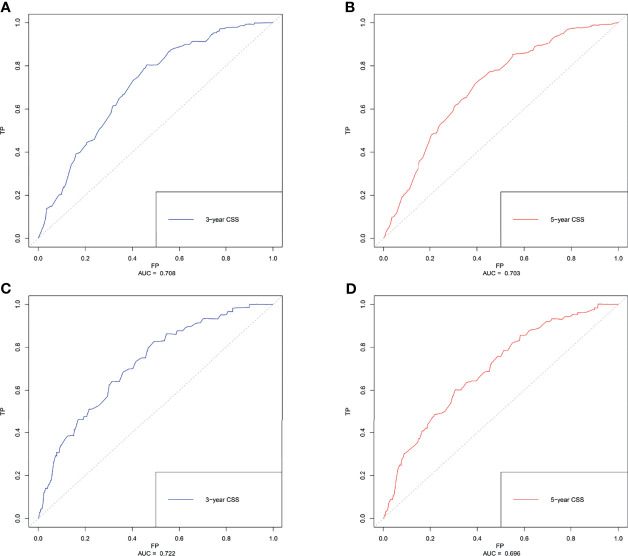
The receiver operating characteristics (ROC) curve and area under the ROC curve (AUC). **(A)** predicting 3- year CSS in the training cohort; **(B)** predicting 5- year CSS in the training cohort. **(C)** predicting 3- year CSS in the validating cohort. **(D)** predicting 5- year CSS in the validating cohort. CSS, cancer-specific survival.

**Figure 5:**
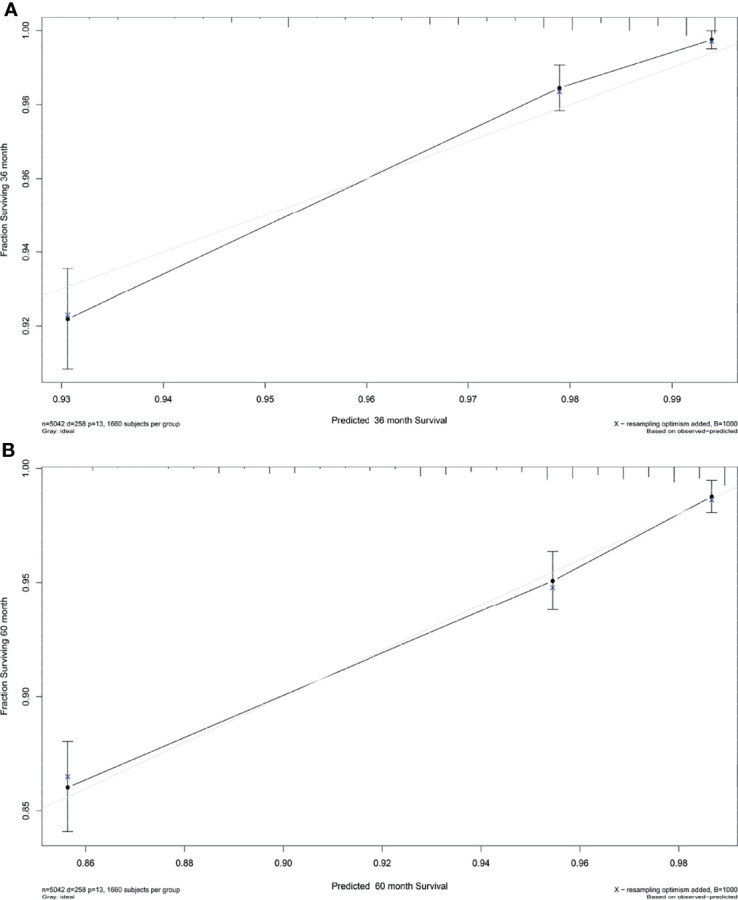
Calibration plot for the prediction of **(A)** 3-year CSS and **(B)** 5-Year CSS in young women with early-stage breast cancer. The solid black line represented the performance of the nomogram, of which the closer fit to the gray line represents the better prediction of the nomogram we constructed. CSS, cancer-specific survival.

Based on the results of multivariate Cox regression analysis, six independent variables including race, tumor location, grade, lymph node status, subtype, and tumor size were screened out for establishing a visualized nomogram to predict the 3- year and 5- year CSS in young women with early-stage breast cancer (**Figure 3**). The model contained a satisfying C-index of 0.783, combined with an AUC of 0.708 in predicting 3- year CSS (**Figure 4A**) and 0.703 in predicting 5- year CSS (**Figure 4B**), respectively. The specific value of each variable was calculated in **Table 3**. Thus, patients could obtain individualized total scores based on their clinicopathological characteristics (race, tumor location, differentiation grade, N stage, molecular subtype, and tumor size) and the corresponding 3-, 5-year CSS probability. Moreover, the accuracy of our nomogram was validated by an internal validation cohort with 2161 cases. The results in the validating cohort also presented good discrimination with an AUC of 0.722 in predicting 3- year CSS (**Figure 4C**) and an AUC of 0.696 in predicting 5- year CSS (**Figure 4D**), respectively. Furthermore, a calibration curve for evaluating the accuracy of the predictive ability in 3- year CSS (**Figure 5A**) and 5- year CSS of young women with early-stage breast cancer was also displayed (**Figure 5B**), which indicated a great agreement in the training data set.

**Table 3:** The specific value of each variable in the nomogram derives from the multivariate Cox logistic regression results.

Characteristics	Score
Race	
Hispanic	18
White	14
Black	34
^ ※^Other	0
Tumor location	
^&^Central	0
Inner	33
Outer	13
^ ¶^other	26
Grade	
well	0
moderately	82
poorly	100
Lymph status	
N_0_	0
N_1_	33
Size	
T_1_	0
T_2_	28
Subtype	
Luminal A	35
Luminal B	0
TNBC	67
Her-2 enriched	34
**Total point for 3-year CSS**	
0.7	299
0.75	291
0.80	281
0.85	269
0.90	252
0.95	224
**Total point for 5-year CSS**	
0.45	300
0.50	294
0.55	289
0.60	283
0.65	276
0.70	269
0.75	260
0.80	251
0.85	239
0.90	222
0.95	194

**^※^**Other: defined as the non-Hispanic Asian/Pacific Islander and American Indian/Alaska Native; **^&^**central: central portion of breast combined with nipple; ^¶^other: axillary and overlapping of the breast. IDC, invasive ductal carcinoma; ILC, invasive lobular carcinoma; IDLC, invasive ductal mixed with lobular carcinoma; TNBC, triple-negative breast cancer; Her-2, human epidermal growth factor receptor-2; T_1_, 0mm≥maximum diameter <20mm; T_2_, 20mm≥maximum diameter <50mm.

## Discussion

Over the past years, with an increasing prevalence of breast cancer worldwide (1–4, 6), the comprehensive and individualized management for patients with this particular disease has gradually attracted much attention, especially in terms of young breast cancer (7, 9, 10, 14, 15). It was believed that age was an independent prognostic indicator among female breast cancer and younger age, especially under the age of 40 years, frequently presented a higher risk of locoregional metastasis, recurrence, and ultimately worse OS outcomes (7, 10, 12). Indeed, while surgery remained to be the first-line strategy in the management of breast cancer, great changes have taken place in terms of the surgical extension (8, 16), especially with the wide application of neoadjuvant chemotherapy (17, 18) and postoperative radiation therapy (19) for early-stage breast cancer. Some scholars even suggested that patients with clinical complete response (cCR) after neoadjuvant chemotherapy might be exempted from the subsequent surgery because there was no significant difference in 5- year OS between patients with cCR and those with pathological complete response (pCR) (20). Furthermore, recent studies have confirmed that breast-conserving surgery would not affect the OS or DFS in young women with early-stage breast cancer, even though it did not improve the long-term survival in this subpopulation like the old population, as compared with mastectomy (8, 16, 21). Additionally, identifying more risk clinical factors and their effects on the prognosis of young women with early-stage breast cancer was equally important for predicting the 3- and 5- CSS. Herein, we retrospectively evaluated the clinicopathological characteristics of young women with early-stage breast cancer from the SEER database.

In our study, six variables including race, tumor location, grade, lymph node status, molecular subtype, and primary tumor size were independent prognostic factors in predicting the CSS of early-stage breast cancer in young women. Specifically, in accordance with the results of previous epidemiological studies, we found young Black women had worse CSS and a higher risk of cumulative incidence, compared with other races. As showed in the results of *Cancer Statistics for Adolescents and Young Adults* (2020 version) (5), young Black women had higher incidence rates of breast cancer than White as well as higher death rates (25.9 vs 22.3 per 100,000 population, 3.9 vs 2.0 per 100,000 population during 2012-2016, respectively). Most recently, Walsh et al. (7) examined a large-scale population of young Black women with breast cancer and they confirmed the young Black women had worse disease-free survival (DFS) than old women, but OS was not significantly different. On the contrary, this divergence of OS or CSS in different races were disappeared among female patients with bone metastasis at presentation (p=0.282, p=0.413, respectively) (22). Interestingly, we determined that the inner location of the primary tumor was associated with relatively poor CSS in young women with early-stage breast cancer (p=0.003). Our finding was consistent with the conclusions of two recent studies from China (23, 24). Yang et al. (24) and Wu et al. (23) both highlighted that lower inner tumor location presented significantly lower DFS or recurrence-free survival (RFS) in young women with early-stage breast cancer. In contrast, central or nipple tumor location was regarded as the risk of axillary lymph node metastasis (25, 26) regardless of the stage of patients at presentation. On the other hand, poorer differentiation grade, triple-negative molecular subtype, lymph node metastasis, and large tumor size have frequently indicated the poor survival of breast cancer (22, 27–29). In our study, we determined similar findings to previous studies (**Table 2**), in which moderately or poorly grade, TNBC subtype, N_1_ status, and T_2_ tumor size all played a pivotal role. Notably, the pathological type in our study comprised a spectrum of IDC (94.2%), ILC (2.2%), and IDLC (3.6%). However, we did not find any significant difference among these subtypes (p=0.431).

Currently, nomograms have been used for conveniently predicting the outcomes of many kinds of cancers (30–33) which significantly improved the process of individualized medical decision-making. Regarding breast cancer, some previous studies have developed survival predicting models for those with N_3_ status, HR-/Her-2-, or IV stage (22, 27, 29, 34). Although those models contained good predictive ability, yet the patients they enrolled have already been in a dangerous situation which should be recommended to elect relatively more aggressive treatment modalities. On the contrary, while young breast cancer often presents a worse prognosis than older patients, whether radical treatments should also be recommended for this subpopulation with early-stage breast cancer was rarely exemplified (28, 35). Therefore, we established the first nomogram for predicting the 3- year, 5- year CSS in young women with early-stage breast cancer. As expected, the nomogram showed sufficient discrimination ability with a C-index of 0.783. The AUC of 3- year CSS and 5- year CSS in both training and validating cohorts were close and even higher than 0.7, which indicated the satisfied prediction capacity. Furthermore, the calibration plot also demonstrated the high agreement of this model.

Admittedly, there were still some limitations that have to be addressed in the future study. First, although the sample size of this study was considerable, the character of the retrospective design was inevitably flawed with bias. Second, some clinical risk factors like Ki-67 index (36, 37) and BRCA1- and BRCA2- related mutation (15, 38) as well as high 21-Gene Recurrence Score (21-GRS) (39) which have been proved to be related to worse OS in patients with breast cancer were unavailable in the SEER database. Third, the detailed information of hormone receptors (estrogen receptor and progesterone receptor) and Her-2 status were unavailable in the SEER database. Thus, further study should be performed to fill this gap and make more accurate calculations to better define the molecular subtype of patients in accordance with the latest guideline (40, 41). Furthermore, we did not investigate the role of adjuvant chemotherapy or radiotherapy in young women with early-stage breast cancer because of the unknown chemotherapy regimens and the scope of radiotherapy in these patients. Fourth, the information of insurance and socioeconomic status which were determined to be associated with the young breast cancer-specific survival in other studies (14, 42) was also missing. Those important variables should be considered in future research and further prospective randomized controlled studies are urgently needed to obtain more detailed strategies on the field.

## Conclusion

In summary, we identified six variables including race, tumor location, differentiation grade, lymph node status, molecular subtype, and primary tumor size were independent prognostic factors in predicting the CSS of early-stage breast cancer in young women. Based on these variables, we successfully established the first nomogram for predicting the 3-, 5- year CSS in this particular group and could help clinicians better distinguish the young breast cancer patients at high risk.

## Data Availability Statement

The original contributions presented in the study are included in the article/supplementary material. Further inquiries can be directed to the corresponding authors.

## Ethics Statemment

This article does not contain any studies with human participants or animals performed by any of the authors.

## Author Contributions

Conception and design: RL, HL, GY, YF. (II) Administrative support: YM, YF. Provision of study materials or patients: YM, YF. Collection and assembly of data: RL, ZX. Data analysis and interpretation: All authors. Manuscript writing: All authors. Final approval of manuscript: All authors.

## Funding

This work was supported in part by the Chongqing Science and Health Joint medical scientific research project (2021MSXM283) and China Postdoctoral Science Foundation funded project (2018M633329) for RL.

## Conflict of Interest

The authors declare that the research was conducted in the absence of any commercial or financial relationships that could be construed as a potential conflict of interest.

The reviewer SL declared a shared affiliation with the authors to the handling editor at time of review.

## Publisher’s Note

All claims expressed in this article are solely those of the authors and do not necessarily represent those of their affiliated organizations, or those of the publisher, the editors and the reviewers. Any product that may be evaluated in this article, or claim that may be made by its manufacturer, is not guaranteed or endorsed by the publisher.
